# Factors influencing cognitive reactivity among young adults at high risk for depression in China: a cross-sectional study

**DOI:** 10.1186/s12889-020-08845-9

**Published:** 2020-05-15

**Authors:** Fei Fei Huang, Zhi Peng Wen, Qi Li, Bin Chen, Wen Jie Weng

**Affiliations:** 1 School of Nursing, Fujian Medical University, No 1 Xueyu Road, Minhou county, Fuzhou, 350108 Fujian China; 2 Neurology Division, the affiliated hospital of Putian University, Putian, China; 3 Neurosurgery Department, 900th Hospital, Fuzhou, Fujian China; 4 Psychiatric Department, Fuzhou Fourth Hospital, Fuzhou, Fujian China; 5 Psychiatric Department, Putian psychiatric Hospital, Putian, Fujian China

**Keywords:** Depression, Cognitive reactivity, Young adults, Survey, China

## Abstract

**Background:**

Understanding the factors influencing cognitive reactivity (CR) may help identify individuals at risk for first episode depression and relapse and facilitate routine access to preventative treatments. However, few studies have examined the relationship between CR and depression in Asian countries. This study was performed to assess the current status of CR among Chinese young adults and explore influencing factors.

**Methods:**

A national cross-sectional online study using convenience sampling was conducted among 1597 healthy young adults in China (response rate: 93.94%) with a mean age of 24.34 (SD = 5.76) years.

**Results:**

The mean CR score was 51.36 ± 18.97 (range 0–130). Binary logistic regression showed that a low level of CR was associated with the following factors: high self-compassion, high social support, high resilience, high monthly household income, and living in a rural area, with odds ratios (ORs) ranging from 0.14 to 0.70. Young adults in full-time employment, experiencing poor sleep, with high neuroticism, who reported frequent sad mood, and who had a high intensity of negative life events had increased CR to depression, with ORs ranging from 1.18 to 6.66. The prediction probability of these factors was 75.40%. Causal relationships among the influencing factors and CR could not be explored.

**Conclusions:**

The self-reported CR levels among Chinese young adults were moderate. Enhancing self-compassion, resilience, and social support for young adults and reducing negative life events, neuroticism, and poor sleep may help decrease CR. These findings may help healthcare providers or researchers determine how to cultivate and improve the CR of young adults by establishing documented policies and/or improving intervention efficacies.

## Background

Depression is one of the most prevalent psychological disorders worldwide and places a significant economic burden on society [1]. In 2017, it was ranked by the World Health Organization (WHO) as the single largest contributor to global disability [2]. Depression has historically been viewed as a condition affecting older adults [3], however, many patients experience their first episode early in life. In recent years, young adults aged 18 to 35 years are increasingly recognized as a population group significantly affected by depression [3]. It is estimated that approximately 25% of young adults experience depressive symptoms and 2.5% of these meet the criteria for a major depressive episode, according to the Diagnostic and Statistical Manual for Mental Disorders (DSM-5) major depressive disorder (MDD) criteria [4].

Despite existing evidence-based treatments for major depression, it remains a chronic and recurrent illness, with 85% of people who experience a single episode experiencing another within 15 years [5]. High rates of depression that remain undetected further enhance its debilitating effects, with over 72.3% of individuals with depression not even being aware of their problem [6]. Globally, young adults are often overlooked, misdiagnosed, or undetected compared to older adults, possibly due to the uncertainties (e.g., initiating the roles and responsibilities of adulthood) young people experience being accompanied by irritability and mood fluctuations [7]. Among those who do not meet the diagnostic criteria for major depression, 10 to 35% experience episodes of low mood or sub-threshold depression that significantly impair their quality of life. These alarming statistics underscore the importance of identifying signs of depression in young adults and identifying those at risk of depression, which may provide clinical targets for depression prevention and early interventions [3].

The etiology of depression is complex, with no single underlying cause. According to Beck’s cognitive model, individuals with depression typically experience cognitive distortions and dysfunctional attitudes and beliefs, which tend to decrease but can be reactivated by dysphoric mood. The ease with which such negative cognitions can be (re-)activated by sad mood states is referred to as cognitive reactivity (CR) [8]. In recent years, a promising line of research has highlighted the role of CR in the development, maintenance, and relapse/recurrence of depressive symptoms or clinical depression (e.g., [9–11]). Thus, the relevance of CR to depression should be further explored.

Understanding CR and its influencing factors may help target individuals at risk for the first episode and relapse of depression and increase access to preventive treatments, decreasing the personal and societal burdens of depressive disorders [5]. Previous studies have shown that CR can be directly and indirectly affected by some socio-demographic and psychosocial factors of mixed clinical/healthy individuals, including body mass index (BMI) for adolescents (weight in kilograms divided by the square of the person’s height in meters [kg/m^2^]) [12], depression status and number of previous episodes for recurrent MDD [13, 14], neuroticism [15], negative life events [9], and rumination [16]. Interestingly, previous studies have mainly concentrated on aspects concerning negative psychological outcomes (e.g., neuroticism) and biological markers of vulnerability to depression [5, 17].

Few groups have examined the association between CR and positive psychological resources (e.g., resilience) [18] that are considered to protect individuals from depression. In the current study, we extended this knowledge of protective factors in depression to include personal factors (e.g., resilience, self-compassion) and interpersonal factors (e.g., social support), and explored the associations between these factors and CR.

The majority of studies on CR have been conducted in western countries, and relatively few have examined either the current status or related influencing factors (especially positive ones) of CR in Asian countries. However, cultural teachings often influence beliefs about the origins and nature of mental illness and shape attitudes towards the mentally ill [19]. CR findings from western countries may not directly apply to the Chinese context. For example, in China, where many cultures value “conformity to norms, emotional self-control, [and] family recognition through achievement,” depression is often stigmatized and seen as a source of shame [19].

The “Healthy China 2030” blueprint was proposed in 2017 by the Chinese government [20] based on four core principles: health priority, reform and innovation, scientific development, and justice and equity. It outlines 13 core indicators to be reported in 2020 and 2030. One was the improvement of mental healthcare and preventative services [21]. To help achieve this target, the dual purpose of this nationwide cross-sectional study was to: (a) describe the current status of CR among Chinese young adults at high risk for depression; (b) identify factors associated with CR.

## Methods

### Participants

Between January 2018 and January 2019, a total of 1700 young adults, aged 18–35 years, were enrolled in the online national survey using a convenience sampling method. The popular Chinese online survey platform Wenjuanxing (http://www.wjx.cn) was used. The survey recruitment information was posted to six administrative regions in China, including the northeast, east, north, south-central, southwest, and northwest areas. First, the link for the online survey was sent to the research partners or friends who reside in six administrative regions by email. Then, they posted this link in different forums (e.g., QQ, WeChat). To excluded participants who have a diagnosis of mental illness, such as MDD, bipolar disorder, psychotic disorders, and others, the participants would be asked whether or not receiving a diagnosis of mental illness from a psychiatrist. The sample size was determined by the subject to item ratio of 5–10:1 [22], and the total number of survey items was 166.

### Measures

#### CR

The modified Chinese version of the Leiden Index of Depression Sensitivity (LEIDS-RR-CV) is a 26-item self-report measure of CR to sad mood [6]. Participants are asked to imagine the last time they felt a mild state of dysphoria, and then to indicate the degree to which a list of statements describes their typical cognitions and behaviors in response to a sad mood. The LEIDS-RR-CV contains 26 items from 5 subscales, including hopelessness/suicidality, acceptance coping, aggression, control/perfectionism, and avoidant coping. All of the items are rated using a 5-point Likert-scale (0 = *not at all* to 4 = *very strongly*). Items are all positively worded for CR, and the total score is obtained by summing the scores of all items. Considering the differences in the numbers of items among the five subscales, the average score for each subscale was calculated. A higher total score indicates stronger CR. In this study, the Cronbach’s α coefficient was 0.95 for the overall scale. Huang et al. identified a cut-off score of 60 for LEIDS-RR-CV to screen for healthy individuals at risk for depression in China [6].

#### Social support

The Multidimensional Scale of Perceived Social Support (MSPSS) is a 12-item self-report scale used to measure perceived social support from family, friends, and significant others [23, 24]. The scale employs a 7-point rating scale ranging from 1 (*very strongly disagree*) to 7 (*very strongly agree*). The total scores of the scale range from 12 to 84, with higher scores indicating greater levels of social support. The social support is classified into low, middle, and high support levels according to the cut-off score ranges of the MSPSS of 12–36, 37–60, and 61–84, respectively [24]. In this study, the Cronbach’s α coefficient was 0.97 for the overall scale.

#### Neuroticism

The Neuroticism Subscale of the Chinese Big Five Personality Inventory (NEO-CBF-PI) is the most comprehensive self-report questionnaire measuring the five dimensions of personality, including neuroticism. The CBF-PI consists of 40 items and has been extensively validated [25]. The 8-item neuroticism subscale of the CBF-PI is rated on a 6-point Likert scale (1–6), with the total score ranging from 8 to 48. Higher scores are indicative of a higher level of neuroticism. Based on previous studies [25], levels of neuroticism are classified into high and low according to a cut-off score of 36 for the CBF-PI. In this study, the Cronbach’s α coefficient was 0.87 for the NEO-CBF-PI.

#### Resilience

The Chinese version of the 14-item Resilience Scale (RS-14) developed by Wagnild and Young is one of the most reliable tools in measuring resilience in various age groups and different conditions [26, 27]. It is composed of 14 items representing the “Personal Competence Factor” and “Acceptance of Self and Life Factor.” Each item is graded from 1 (*strongly disagree*) to 7 (*strongly agree*). Graded items are summed to provide a total score, with lower scores indicating less resilience. According to the cut-off value of 74, resilience levels are classified into high and low [27]. The RS-14 has satisfactory internal consistency with Cronbach’s α ranging from 0.87 to 0.91, and stability with test-retest reliability ranged from 0.65 to 0.84 [26]. In this study, the Cronbach’s α of the RS-14 was 0.96.

#### Self-compassion

The Self-Compassion Scale (SCS) is the most commonly used scale to measure self-compassion at times of perceived difficulty [28]. It is composed of 26 items and 6 subscales, including self-kindness, self-judgment, common humanity, isolation, mindfulness, and over-identification. Each item rated on a 5-point Likert-type scale for frequency (1 = *almost never*; 5 = *almost always*). The total score is calculated with the average of the individual subscales, and all negatively scored items are transformed. Levels of self-compassion are classified high and low according to 75% of the total SCS scores (130*0.75) as based on a previous study [29] (i.e., a score of 98). In this study, the Cronbach’s α of the SCS was 0.77.

#### Life events

The 48-item Life Events Scale (LES) is used to evaluate negative and positive life events that have occurred during the previous year or longer, including family, work or study, and social events [30]. Each of the 48 life event items was anchored to 4 questions: (i) when it happened, measured by “*never,*” “*in the past 1 month,*” “*in the past 1 year or longer*”; (ii) whether it was positive or negative for the target person; (iii) the impact on the target person’s mental health, measured by a 5-point scale ranging from “*no impact*” to “*very severe impact”*; (iv) the duration of the event, measured by a 4-point scale ranging from “*3 months,*” “*6 months,*” “*≤1 year*” to “*longer.*” The intensity score of each life event is calculated by the time when it happened (i) multiplied by the duration (iii) and impact (iv). The total intensities of positive and negative life events are summed by the intensity score of each positive or negative life event. Based on the 75% value of this score [29], the total intensities of positive and negative life events are further classified into high and low levels. In this study, the Cronbach’s α of the LES was 0.94.

#### Socio-demographic and clinical characteristics

Participants were asked about their socio-demographics including residential area (northeast/ eastern/ north/ south central/ southwest/ northwest area), residential location (urban/suburban/rural), age, sex (male/female), marital status (married/ unmarried/ others), educational level (less than high school degree/high school degree /bachelor’s degree or higher), religious belief (no/ yes), monthly household income (Yuan, RMB)(< 1000/1000–2999/3000–4999/5000+), employment status (students/ full-time employment/ unemployment/ farmer/ other), and living mode (living by oneself/ living with spouse/ living with family/ others).

We also collected clinical characteristics including smoking status(yes/no), BMI, family history of mental illness (no/yes/unclear), whether they had previously experienced depression (yes/no), the frequency of sad mood in the past month (none/ occasionally/ sometimes/ often/ always), and their sleep quality (very good/ good/ average/ bad/ very bad). In this study, according to the WHO-recommended BMI cut-off values for adults [31], BMI is classified as underweight, BMI < 18.5  kg/m^2^; normal weight, BMI 18.5 to 25.0  kg/m^2^; and overweight, BMI ≥25.0 kg/m^2^.

### Procedure

All procedures were approved by the ethical committee of Fujian Medical University (No. FMU2017024), and informed consent was obtained from all participants. The study adhered to the STROBE (Strengthening the Reporting of Observational studies in Epidemiology) statement [32]. All measures were completed via the Wenjuanxing platform. The participants read and signed the written informed consent form on the platform before they completed the questionnaire in approximately 20–30 min. The questionnaire could not be submitted if it was less than half complete or contained repeated answers. Participants who completed the survey were remunerated with a RMB 10 gift card.

### Statistical analysis

Data analyses were conducted using SPSS 24.0 (IBM, Armonk, NY, USA). Approximately 5% of missing data were replaced using mean value substitution, and *p* < 0.05 was considered statistically significant. The data met the assumptions of normality as one-sample Kolmogorov-Smirnov tests were not statistically significant. Continuous variables are expressed as means and standard deviations (SDs) and were dichotomized to improve comparison [33]. Categorical variables are expressed as proportions or percentages.

Young adults with a LEIDS-RR-CV total score < 60 were considered the normal group (NG), while those with a score ≥ 60 were considered the risk for depression group (RDG).

We performed three analysis steps to identify influencing factors of CR. First, chi-square tests were used to compare the differences in socio-demographic and clinical variables, self-compassion, resilience, social support, neuroticism, and life events between the two groups. Second, the collinearity of the independent variables was examined by the variance inflation factor (VIF) before conducting binary logistic regression. The VIFs of the 11 variables ranged from 0.45–2.63 (ideal is < 4.0), suggesting no violations of the regression assumptions [34].

Third, binary logistic regression with a forward conditional method was conducted to determine the influencing factors associated with CR. There is evidence in the logistic regression literature that backward selection is often less successful than forward selection because the full model fit in the first step is the model most likely to result in a complete or quasi-complete separation of response values [35]. The dependent variable was whether the young adults were at risk for depression. The variables that were statistically significant in chi-square tests were input as independent variables.

## Results

A total of 1597 valid questionnaires were returned out of the 1700 questionnaires distributed (response rate, 93.94%). Specifically, the response rate for the northeast, east, north, south-central, southwest, and northwest areas was 92.85, 91.66, 96.74, 95.84, 91.83, 94.73%, respectively. The mean age of young adults was 24.34 years (SD = 5.76), and the average BMI was 22.21 kg/m^2^ (SD = 6.54). Table 1 shows the socio-demographic characteristics of all participants.

**Table 1:** Socio-demographic and clinical characteristics comparison of the NG and RDG

Variables	Total%	NG (*n* = 1103) n%	RDG (*n* = 494) n%	χ2	*p*
Residential area				17.65	< 0.001
Northeast area	12.52	120 (10.89)	80 (16.19)		
East area	37.13	460 (41.70)	133 (26.92)		
North area	14.84	127 (11.51)	110 (22.27)		
South-central area	11.77	118 (10.70)	70 (14.17)		
Southwest area	10.46	117 (10.60)	50 (10.12)		
Northwest area	13.27	161 (14.60)	51 (10.33)		
Sex					
Male	22.79	241 (21.85)	122 (24.70)	2.43	0.30
Female	77.21	862 (78.15)	372 (75.30)		
Residential location					
Urban	50.22	524 (47.51)	278 (56.28)	12.41	< 0.001
Suburban	10.90	118 (10.70)	56 (11.34)		
Rural	38.89	461 (41.79)	160 (32.38)		
Religious belief					
No	83.47	926 (83.95)	407 (82.39)	0.73	0.39
Yes	16.53	177 (16.05)	87 (17.61)		
Education level					
Less than high school degree	4.44	50 (4.53)	21 (4.25)	4.51	0.11
High school degree (including technical training)	10.71	130 (11.79)	41 (8.30)		
Bachelor’s degree or higher	84.85	923 (83.68)	432 (87.45)		
Monthly household income (yuan, RMB)					
< 1000	24.05	341 (30.92)	43 (8.70)	14.92	< 0.001
1000–2999	20.16	184 (16.68)	138 (27.94)		
3000–4999	28.18	310 (28.11)	140 (28.34)		
5000+	27.61	268 (24.29)	173 (35.02)		
Employment status					
Students	41.08	490 (44.42)	166 (33.60)	25.44	< 0.001
Full-time employment	50.09	513 (46.51)	287 (58.10)		
Unemployment	0.88	6 (0.55)	8 (1.62)		
Farmer	0.69	7 (0.63)	4 (0.81)		
Other (e.g., retired, homemaker)	7.26	87 (7.89)	29 (5.87)		
Marital status					
Married	35.38	382 (34.63)	183 (37.04)	2.14	0.71
Unmarried	63.12	705 (63.92)	303 (61.34)		
Others (e.g., divorced, widowed)	1.5	16 (1.45)	8 (1.62)		
Family history of mental illness					
No	90.61	1034 (93.74)	429 (86.84)	25.17	< 0.001
Unclear	7.58	65 (5.90)	56 (11.34)		
Yes	0.81	4 (0.36)	9 (1.82)		
Living mode					
Living by oneself	17.66	208 (18.85)	74 (14.98)	4.96	0.17
Living with spouse	13.21	177 (16.05)	34 (6.88)		
Living with family	45.96	507 (45.97)	227 (45.95)		
Others	23.17	211 (19.13)	159 (32.19)		
Smoking status					
Yes	8.14	84 (7.62)	46 (9.31)	1.73	0.42
No	91.86	1019 (92.38)	448 (90.69)		
The frequency of sad mood in the past month					
None	12.46	168 (15.32)	31 (6.27)	111.51	< 0.001
Occasionally	54.35	639 (57.93)	229 (46.36)		
Sometimes	22.67	230 (20.85)	132 (26.72)		
Often	9.20	53 (4.82)	94 (19.03)		
Always	1.31	13 (1.18)	8 (1.62)		
Sleep quality					
Very good	27.80	345 (31.28)	99 (20.04)	65.30	< 0.001
Good	32.74	381 (34.54)	142 (28.74)		
Average	31.68	323 (29.28)	183 (37.04)		
Bad	6.95	49 (4.45)	62 (12.55)		
Very bad	0.69	5 (0.45)	8 (1.63)		
BMI (kg/m^2^)					
< 18.5 (underweight)	16.22	181 (16.41)	75 (15.18)	2.29	0.32
18.5–24.9 (normal weight)	64.93	723 (65.55)	314 (63.56)		
≥ 25 (overweight)	19.04	199 (18.04)	105 (21.26)		
Self-compassion					
Low	90.11	989 (89.66)	450 (91.09)	45.24	< 0.001
High	10.21	114 (10.34)	49 (8.91)		
Resilience					
Low	51.70	560 (50.77)	266 (53.85)	13.70	< 0.001
High	48.30	587 (49.23)	184 (46.15)		
Social support					
Low	12.60	151 (13.70)	51 (10.32)	69.30	< 0.001
Middle	44.40	433 (39.26)	270 (54.67)		
High	43.00	563 (51.04)	129 (126.11)		
Neuroticism					
Low	94.18	1075 (71.50)	429 (86.84)	70.16	< 0.001
High	5.82	28 (30.10)	65 (13.16)		
Intensity of positive life events					
Low	81.09	944 (85.58)	351 (71.05)	46.99	< 0.001
High	18.91	159 (14.42)	143 (28.95)		
Intensity of negative life events					
Low	68.25	811 (73.53)	279 (56.48)	45.77	< 0.001
High	31.74	292 (26.47)	215 (43.52)		

*Note:* Abbreviations: *BMI* body mass index, *NG* normal group, *RDG* risk for depression group

### CR scores of the young adults

The participants were classified into two NG and RDG according to the LEIDS-RR-CV cut-off score. In total, there has 494 young adults (30.93%) in RDG, and 1103 young adults (69.07%) in NG. As shown in Table 2, the total mean scores for the LEIDS-RR-CV in Chinese young adults were 51.36 ± 18.97 (range 0–130) overall, 41.30 ± 11.80 (range 0–59) for the NG, and 73.82 ± 11.07 (range 60–130) for the RDG. The highest and lowest mean scores were for avoidant coping (2.24 ± 0.86) and hopelessness/suicidality (1.73 ± 0.93), respectively. Furthermore, there were statistically significant differences in the total and sub-scale scores between the NG and RDG (see Table 2).

**Table 2:** Scores for cognitive reactivity among Chinese young adults

Variables	Total(*n* = 1597)			NG (*n* = 1103)		RDG (*n* = 494)		*t* value
	Total score	Average score	Item No	Total score	Average score	Total score	Average score	
ACC	7.92 ± 3.17	1.98 ± 0.79	4	7.02 ± 2.89	1.76 ± 0.72	9.90 ± 2.84	2.48 ± 0.71	−18.40^*^
CTR	10.32 ± 3.96	2.06 ± 0.79	5	8.46 ± 2.87	1.69 ± 0.57	14.46 ± 2.74	2.89 ± 0.55	−40.77^*^
AGG	8.78 ± 4.39	1.76 ± 0.88	4	6.67 ± 2.84	1.67 ± 0.57	13.48 ± 3.51	3.37 ± 0.50	−38.50^*^
AVC	15.69 ± 6.00	2.24 ± 0.86	7	12.75 ± 4.13	1.82 ± 0.59	22.25 ± 4.02	3.18 ± 0.57	−44.09^*^
HOP	8.66 ± 4.65	1.73 ± 0.93	5	6.39 ± 2.99	1.28 ± 0.60	13.72 ± 3.57	2.74 ± 0.71	− 40.49^*^
Total score	51.36 ± 18.97	1.98 ± 0.73	26	41.30 ± 11.80	1.59 ± 0.45	73.82 ± 11.07	2.84 ± 0.43	−53.53^*^

*Note:* Average score = total score/numbers of items; * *p* < 0.01

Abbreviations: *ACC* acceptance coping, *AGG* aggression, *AVC* avoidant coping, *CTR* control/perfectionism, *HOP* hopelessness/suicidality, *NG* normal group, *RDG* risk for depression group

### Factors associated with CR in young adults at risk for depression

As shown in Table 1, the differences in residential area and location, monthly household income, employment status, family history of mental illness, frequency of sad mood in the past month, and sleep quality between groups were all statistically significant (*p* < 0.05). Table 1 also shows that young adults in the NG had higher levels of self-compassion, resilience, and social support and had experienced more positive life events than those in the RDG (*p* < 0.05). The levels of neuroticism and negative life events for young adults in the RDG were higher compared to individuals in the NG (*p* < 0.05).

The predictors of CR determined using binary logistic regression are shown in Table 3. The analysis indicated that the main predictors influencing CR were high levels of self-compassion, followed by residence in a rural location, having high social support, resilience, and high monthly household income. For example, participants with high self-compassion were 0.14 times likely to report CR to depression than individuals with low self-compassion (odds ratio [OR] = 0.14, 95% confidence interval [CI] 0.05–0.71).

**Table 3:** The significant predictors of cognitive reactivity among Chinese young adults at risk for depression

Variable	OR/Exp(B)	*p*-value	95% CI
Residential location			
Urban	Ref		
Rural	0.54	0.04	0.34–0.85
Employment status			
Students	Ref		
Full-time employment	2.54	< 0.01	1.46–4.40
Monthly household income(yuan)			
< 1000	Ref		
5000+	0.70	0.03	0.50–0.97
Frequency of sad mood in the past month			
None	Ref		
Always	4.29	0.04	1.96–9.08
Sleep quality			
Very good	Ref		
Bad	1.18	0.04	1.03–1.92
Social support			
Low	Ref		
High	0.58	< 0.01	0.43–0.78
Self-compassion			
Low	Ref		
High	0.14	0.001	0.05–0.71
Neuroticism			
Low	Ref		
High	6.66	< 0.01	1.41–3.33
Resilience			
Low	Ref		
High	0.63	< 0.01	0.47–0.86
Intensity of negative life events			
Low	Ref		
High	1.49	0.01	1.12–2.00

*Note:* Abbreviations: *CI* confidence interval, *OR* odds ratio, *Ref* reference

In contrast, young adults with a high level of neuroticism, followed by those with frequent sad mood in the past month, in full-time employment, having a high intensity of negative life events, and bad sleep quality had higher CR to depression. For example, the risk of CR to depression in young adults with higher neuroticism was 6.66 times higher than for those with lower neuroticism (OR = 6.66, 95% CI 1.41–3.33). The overall prediction probability of these factors was 75.40%.

## Discussion

The psychological well-being of young people has became a public health concern worldwide [36]. This population is vulnerable to psychological distress, and in particular, depression. Screening at-risk populations and providing targeted measures are cost-effective strategies to prevent and treat depression [6].

To our knowledge, this is the first study to explore the relationship between CR and depression and the associated influencing factors in young adults in China. The results revealed moderate levels of self-reported CR among Chinese young adults. Although the same instrument was used (LEIDS), our findings were still higher than in a population of mixed clinical/healthy individuals in the Netherlands [37] and a Spanish mixed-population [38], and slightly lower than non-depressed Iranian individuals [39] and recurrently depressed patients in remission in the Netherlands [5]. These disparities could be attributable to sociocultural aspects differences and the utilization of or access to mental health services differences for Asian and western countries, which needed to be further explored [19].

In this study, the CR levels of Chinese young adults were mainly reflected in terms of avoidant coping and control/perfectionism, both of which are closely linked to depression. For example, the highest score for avoidant coping indicates that when young individuals were under stress, they generally applied a maladaptive coping mechanism characterized by cognitive and behavioral efforts to deny, minimize, or avoid dealing with the situation. Although previous research has implicated CR both in the first episode and relapse of depression, the influencing factors or predictors of CR were unknown. Our findings provide the first evidence regarding factors associated with reduced or increased CR in Chinese young adults.

### Factors associated with reduced CR

In our study, self-compassion and resilience were negatively associated with CR. Kuyken et al. also found an association between CR and self-compassion skills [40]. This may be due to self-compassion and resilience both being important protective factors against depression. Higher levels of self-compassion and resilience are typically related to greater psychological health, demonstrated through lower levels of depression and anxiety [36, 41]. In other words, young individuals with higher self-compassion and resilience might be more likely to hold their feelings of suffering with a sense of warmth, connection, and concern, while negotiating, managing, and adapting to significant sources of stress and trauma [36].

Consistent with a previous study [42], we also found that lack of social support was significantly associated with CR. Support from family members, friends, or other people is particularly important for those experiencing stress [42]. This may explain why the level of CR among young adults with stronger social support was significantly lower than those with weaker social support.

As Zeng and Jian reported [43], the prevalence of depression can be directly and indirectly affected by socio-economic status and inequality among different residential locations in China. Our study also found relationships between monthly household income, residential location, and CR with depression. Compared with young adults living in rural areas, CR was higher in young adults living in urban areas. Generally, people residing in cities experience higher stress and more challenges. If they cannot mitigate these, they become more prone to dysfunctional attitudes and depression. Furthermore, our study further confirms and adds to the growing body of evidence that poor economic conditions are strongly link with greater depressive symptoms [44]. In this study, individuals with a high monthly household income had lower CR, suggesting that the risk of depression among young adults may be decreased by reducing the psychosocial stress associated with financial hardship [44].

### Factors associated with increased CR

In line with other studies [15, 45], we also found that neuroticism and poor sleep quality were positively correlated with CR in Chinese young adults. In other words, individuals with higher neuroticism and poorer sleep quality were more likely to respond to mildly negative moods by reactivating thoughts relating to hopelessness (or other negative states), which are in turn, related with depression. Our analysis also revealed that CR among young adults was associated with an increased frequency of sad mood in the past month. The current study also confirms the association between negative life events and the prevalence of depression; when young adults encounter negative life events, they report less happiness and optimism, which may lead to poorer mental health [32]. Employment status was also identified as a risk factor associated with increased CR. That is, young adults in full-time employment encountered greater work-related stress or propensity to depression than students [7, 29].

To effectively reduce the prevalence of depression, healthcare providers need to pay attention to factors associated with reduced or increased CR, which is an important predictor of an episode of depression. By being aware of these potentially influencing factors on CR, we can take targeted measures to reduce CR among young adults by enhancing their self-compassion, resilience, and social support; improving the quality of their sleep; and decreasing their neuroticism and experience of negative life events. For example, employing mindful intervention strategies to decrease neuroticism and problems with emotion regulation. We should pay special attention to young adults with high levels of CR, who reside in urban areas, have low monthly household income, and are employed full time.

### Limitations

Although this study revealed important findings, the results should be interpreted in the context of its limitations. First, the convenience sampling method and recruitment of non-clinical young adults may impact the generalization of the findings. Moreover, the mental illness diagnosis was self-reported by participants, which may have affected its accuracy. Second, the subjectivity associated with the use of self-reported questionnaires may also pose limitations, and our findings should be confirmed by objective measurements in the future, such as clinician-administered assessments. Third, the underlying causal relationships and effect mechanisms between the risk factors and CR were not examined. Future longitudinal or interventional research is needed to address this important issue.

## Conclusions

The self-reported CR levels in Chinese young adults were moderate. Self-compassion, resilience, social support, and high monthly household income were identified as factors associated with lower CR, while negative life events, neuroticism, and poor sleep quality were associated with higher CR. Young adults who were residents in urban areas, in full-time employment, and frequently experienced sad mood were at risk for high levels of CR. These findings may help healthcare providers and/or researchers design interventions to cultivate resilience and improve the CR of young adults.

## Data Availability

The original data are available on request to the corresponding author, after the manuscript published. Whatever, we also considered to provide the original data in public repositories.

## Abbreviations

BMI: Body mass index
CI: Confidence interval
CR: Cognitive reactivity
LEIDS-RR-CV: Chinese version of the Leiden Index of Depression Sensitivity
LES: Life Events Scale
MDD: Major depressive disorder
MSPSS: Multidimensional Scale of Perceived Social Support
NEO-CBF-PI: Neuroticism Subscale of the Chinese Big Five Personality Inventory
NG: Normal group
OR: Odds ratio
RDG: Risk for depression group
RS-14: Chinese version of the 14-item Resilience Scale
SCS: Self-Compassion Scale
VIF: Variance inflation factor
WHO: World Health Organization
